# Unveiling Implicit Collective Memory: Images of Ukraine in Dmitrij Kapitelman’s Novel *Eine Formalie in Kiew*

**DOI:** 10.1080/00787191.2024.2437873

**Published:** 2025-03-26

**Authors:** Aigi Heero

**Affiliations:** Tallinn University

**Keywords:** Kapitelman, Ukraine, Collective memory, Implicit collective memory, Transnational literature, German-Russian relations, War in Ukraine

## Abstract

In the exploration of Dmitrij Kapitelman’s novel *Eine Formalie in Kiew*, this article focuses on issues of identity, memory, and perception through an analysis of the protagonist Dima’s journey. It examines the evolution of Dima’s perspectives on Ukraine, revealing a transformation from a blend of German and post-Soviet viewpoints to a nuanced fusion of perceptions. Thereby, the study discusses the implicit collective memory as described by Astrid Erll, embedded in societal thought patterns and its influence on personal perspectives.

## Dmitrij Kapitelman and his Novels

In the wake of the full-scale Russian invasion of Ukraine in 2022, Europe, and particularly Germany, experienced a profound paradigm shift in its collective consciousness. The ensuing public discourse surrounding the ‘War in Europe’ following Russia’s aggression not only reshaped current public debates but also intertwined with historical memory discourses, such as discussions on the World War II and the post-Soviet time. Significantly, this socio-political upheaval deeply influenced the artistic and literary spheres in Germany. A notable trend emerged wherein numerous German authors with Ukrainian heritage actively voiced their political opinions and unwavering support for Ukraine since the outbreak of the conflict. Particularly poignant are the narratives of authors who found themselves in exile as war refugees (e.g. Kateryna Mishchenko) or acted as advocates for those left behind in Ukraine, including friends and family members (e.g. Katja Petrowskaja or Serhij Zhadan). Through their literary expressions, they infused an existential dimension into the public discourse, prompting critical inquiries into German and post-Soviet memory discourses.^1^

The confluence of geopolitical events and literary expression has paved the way for a nuanced exploration of collective memory and the complex interplay between personal narratives and historical events. According to Egger, a noticeable trend has already emerged in recent years, wherein activist literature actively seeks public platforms, utilizing formats such as essays, feature articles, panel discussions, and social media posts^2^; this trend is amplified by the Eastern European turn^3^ and driven by the hope that *Zeitenwende*^4^ will have a profound impact on Germany’s public opinion on a wider scale (which has not really happened yet).^5^ While not solely the product of Ukrainian exiles or Eastern European writers who had already diversified German-language literature prior to the Ukrainian conflict, their contributions significantly drive this shift.^6^ This process places contemporaneity and political engagement at the forefront of both literary and public interest.

One prominent author contributing to this evolving literary landscape is Dmitrij Kapitelman. Born in Kyiv in 1986, Kapitelman’s familial roots trace back to a Jewish-Ukrainian heritage through his father, Leonid Kapitelman, and his Moldovan mother, Vera Romashkan. Notably, Kapitelman adopted his mother’s surname as a precaution against the pervasive anti-Semitic sentiments prevalent in the Soviet Union during that era.^7^ At the age of eight, Kapitelman immigrated to Germany with his family as a quota refugee.^8^ He pursued his education, studying political science and sociology at the University of Leipzig, later graduating from the German School of Journalism in Munich. Presently residing in Berlin, Kapitelman leads a multifaceted career as a writer, freelance journalist and musician. His literary corpus comprises two novels, a collection of articles spanning various media platforms and numerous interviews. Kapitelman’s candid exploration of contemporary German political dynamics^9^ is emblematic of his unreserved engagement with socio-political discourse. Furthermore, he actively harnesses the potential of social media to express his support for Ukraine. For instance, in early 2022, he criticized Germany’s defensive stance and reluctance to supply arms to Ukraine.^10^

His first novel, *Das Lächeln meines unsichtbaren Vaters*, was published in 2016. This work has been discussed as a ‘border-crossing novel’, depicting a journey of a father and a son who travel from Germany to Israel and showing how national, cultural, and religious border crossings may have an effect upon a person’s self-conception.^11^ Furthermore, the novel examines how the main character, Dima, re-interprets his Jewish-Ukrainian family history, which facilitates his transition from a migrant background and non-religious Jewish affiliation to become a German, a Jew, and, finally, a German Jew.^12^ It should be pointed out that, even though in this novel, references to Judaism and Jewishness abound, Holocaust memory is rather not part of the narrative.^13^ Therefore, Dima’s new identity as ‘German Jew’ can be considered rather secular and cosmopolitan, a feature revived also by other post-Soviet authors.^14^

In the follow-up novel *Eine Formalie in Kiew*,^15^ published in 2021, Kapitelman delves further into the complexities of identity, both individual and collective. Through the lens of the protagonist Dima and his familial ties, the novel offers a critical examination of the migrant experience in the modern Germany. The novel’s style adds depth to its portrayal: through subtle linguistic devices, such as wordplay, irony, and juxtaposition, Kapitelman creates a rich narrative that allows readers to engage with the text on multiple levels. As noted by Koreneva and Ludewig, particularly noteworthy in Kapitelman’s novel is the use of the ethnolect, characterized by a mixture of German and Russian expressions, wordplay, unusual phraseology, and intercultural references.^16^

In this novel, Dima has decided to apply for German citizenship, as he came to Leipzig in 1994, went to school, and studied there. In his own words, he is employed, pays his taxes punctually, and is ‘verfassungspatriotisch’ (constitutionally patriotic) (8).^17^ However, one of the other reasons for Dima’s decision might have been the continually difficult and tense relationship between him and his parents, especially with the mother, Vera (28).^18^ Thus, Dima aspires to create emotional distance from his parents and disengage from the intricacies of his family’s historical background which is also connected to difficult childhood memories to Kyiv and Ukraine before the departure to Germany.

To complete the application process, Dima needs a renewed birth certificate, issued and apostilled in Kyiv, Ukraine. This takes him on a journey back to Ukraine, which he left in 1994 and has not visited ever since. On this journey, he deals with the Ukrainian public authorities, but he also revisits known places and meets old friends. Thereby, more and more memories of his childhood resurface. Furthermore, he re-establishes the connection to his Ukrainian family as well as to his parents who later also come to Kyiv. The more time goes, the more Dima rediscovers his Ukrainian roots and learns to accept his origin.

In terms of literary genres, this novel blends elements of a *Generationenroman*, focusing on the dynamics across multiple generations within a family, with those of a *Bildungsroman*, depicting the protagonist’s journey of personal growth and self-discovery. Through Dima’s evolving relationships with his parents and his own inner development, the novel provides readers with an exploration of generational dynamics and the transformative power of lived experiences.

While Kapitelman’s novel can be situated within the broader framework of the transnational German-language literature,^19^ or more specifically, Eastern Turn literature, it is essential to approach this classification with critical distance, considering the reservations expressed by some authors, regarding the labelling of authors based on their origin.^20^ Nonetheless, there are some similarities between Kapitelman’s work and the themes explored within the Eastern Turn literature. Like many Eastern Turn authors, Kapitelman’s novel engages in memory work, reflecting on the experiences of younger years in the Soviet Union and simultaneously, depicting the complexities of a migrant life in Germany. The novel incorporates cultural comparisons between the protagonist’s country of origin and his new home, mirroring the stylistic elements found in other Eastern Turn works, such as inventive use of the German language, humour in depicting the communist past, nostalgic recollections of pre-1989 childhood and the use of cultural stereotypes to illustrate Western adaptation.^21^

Such narratives, as stated by Rothberg, may generate novel forms of memory through migration, shaping the recollections of both migrants and the residents of the destination country. Rothberg suggests that migration creates a ‘thickening’ of place, blending histories and memories to form new cultural connections. This concept refers to the presence of diverse transnational layers of memory, allowing individuals to incorporate existing memories and stories into their own unique narratives.^22^ In this context, Kapitelman’s novel invites readers to critically engage with entrenched cultural stereotypes, particularly those surrounding Ukraine. This exploration of stereotypes aligns with observations by Haines, who notes that authors from Eastern backgrounds may strategically employ cultural stereotypes and humour as literary devices in their writing.^23^ In Kapitelman’s novel, the narrator occasionally assumes the role of a self-appointed mediator, humorously explaining aspects of Eastern European life with a pseudo-scientific tone. For instance, he describes kvas as a popular drink in Eastern Europe, adding that the tastiest ones are typically sold by toothless grandmothers in floral aprons, directly from the barrel (71). Thereby, cultural clichés are intentionally utilized: not only to entertain readers but also to expose and challenge these clichés and national stereotypes.^24^

Thus, the present study focuses on different perceptions of Ukraine represented by various characters, including Dima himself. A post-Soviet perspective and somewhat nostalgic attitude towards Soviet Ukraine is presented by Dima’s mother, Vera, and other older characters. In contrast, a German view of Ukraine is represented by Mrs. Kunze, the clerk in the foreigners’ registration office of Leipzig, and the German border guards. According to this view, Ukraine is an almost undeveloped country, afflicted with high levels of corruption and populated by people who constantly need lecturing and guidance on how to live a proper life. However, young Ukrainians in Kyiv, such as Rostik, Dima’s friend, and Zoja and Andrej, Dima’s cousins, are critical of Ukrainian politics but believe in the country’s future. Manoeuvring between these differing opinions, Dima realizes that he has several clichés about Ukraine in his mind, primed by his parents’ negative memories about Kyiv during the Soviet era and mediated by German media. By the end of his travels, he is finally able to form an image of Ukraine himself and accept that he is a ‘German Jew’ originally from Ukraine. In light of the Russian full scale invasion of Ukraine in February 2022, *Eine Formalie in Kiew* is particularly relevant in raising important questions about how mediated images can define our attitudes towards other countries and societies.

## Dima’s Perception of Ukraine before and at the Beginning of his Travel

Dima has not visited Ukraine since he left in 1994. Therefore, his perception of Ukraine is strongly influenced by his parents’ view. Vera and Leonid lived in the Soviet Union, and their view is, understandably, shaped by the memories of this period of life. Moreover, Dima unconsciously adopts the perception of Ukraine arbitrated by German media.

Hereby, Astrid Erll’s notion of ‘implicit collective memory’ might be applied. This concept delves into the subtle ways in which past events influence the present, often without individuals being fully aware of it.^25^ According to Erll, these influences manifest in various forms, such as narrative structures, stereotypes, framing patterns, and worldviews, which are ingrained in society and passed down through generations. These elements are typically not explicitly recognized or discussed but play a significant role in shaping how people perceive the cultural and historical context they are living in.^26^ Also schematization, as outlined by Erll, becomes a potent tool in the transmission of implicit collective memory. This process involves the utilization of visual symbols, subconscious master narratives, cultural stereotypes, metaphors, values, norms, and specific behavioural patterns. By employing these elements, societies unconsciously transmit collective memories, contributing to the shaping of individual and group identities.^27^ For example, when considering a nation’s collective memory of a historical event, implicit collective memory could involve subconscious narratives or stereotypes about heroes, villains, or national identity that subtly influence how people perceive current events, political figures, or even neighbouring countries. These implicit memories, transmitted or ‘*mediated* and *remediated*’ through cultural symbols, literature, or media, impact public attitudes and behaviours, showcasing the subtle yet powerful ways in which historical narratives continue to shape contemporary society.^28^

For instance, in a reflective essay published in 2022, Kapitelman recounts his journey to Crimea in 2019. He candidly acknowledges that at that time, he may have been influenced by a ‘westverblendet’ (West-blinded) perspective as he struggled to comprehend the cowering of people before the Russian border guard or their nonchalant behaviour on the beaches of Sevastopol being unconcerned about the looming threat of an industrial oil leak — phenomena that appeared strange seen from his ‘Western’ perspective. However, by 2022, he attested to a transformative shift in his viewpoint, signifying a departure from the earlier Western-centric lens through which he had previously interpreted his experiences.^29^

Similarly, Dima’s perception of Ukraine in *Eine Formalie in Kiew* before his travel seems to be influenced by German media and people’s opinions. This perspective is first introduced in this novel by Mrs. Kunze, a co-worker at the foreigners’ registration office in Leipzig who is the responsible official for Dima’s application process. She explains to Dima (in pure Saxon dialect) that he needs, in addition to all other documents, a renewed birth certificate which can only be issued and apostilled in Kyiv. As Dima reacts distressed to this information, she comments: ‘Joa, üm den Umgang mid den ukrainsch’n Behörd’n beneide ich Sie ooch nisch. Dar gibt’s immor bsönders viele Üngereimdhaiden.’ (14) The use of the dialect contributes to the humour of the scene by introducing a linguistic contrast and highlighting the absurdity of the bureaucratic conversation. The exaggerated language like ‘bsönders viele Üngereimdhaiden’ amplifies the comedic effect. Thus, Mrs. Kunze refers to a common stereotype about bureaucratic inefficiencies in post-Soviet countries and sees Ukraine as a corrupted state with giant bureaucratic machinery and does not have much trust in Ukrainian officials.

According to Schumann and Ivanov, in Germany, the picture of Ukraine is interspersed with stereotypes and general ascriptions.^30^ Ukraine is, in German media, mostly perceived as a country of permanent crisis and war, dominated by political and economic stagnation, sluggish reform efforts, and corrupt and unreliable decision-makers.^31^ It is seen as part of Russia or the former Soviet Union, sometimes even as a radical right-wing, nationalistic state, which adopted a European perspective only for Euromaidan.^32^ An explanation of this point of view could be the Russian media and propaganda campaign in 2014–2015, when Russia successfully managed to spread disinformation.^33^ In relation to that, it is possible to speak about framing, that is, the process by which the media places the events and topics they report in a certain perspective or structures narratives due to the way the stories are told and retold.^34^ For instance, before the Russian invasion in 2022, German media seemingly drew a rather reduced picture of Ukraine, which mostly appeared to be crisis-driven,^35^ reflecting a common media frame that emphasized conflict, instability, and geopolitical tensions. This portrayal aligns with Erll’s emphasis on media frames, which not only shape how the past is collectively remembered but also exert a forward-facing influence by framing current events. However, the true influence of these media frames lies in their subconscious effects on the audience’s reception and perception, shaping their understanding of complex geopolitical situations and influencing their attitudes and opinions.^36^

At the beginning of the novel, both Mrs Kunze and Dima are quite content with a schematic picture of Ukraine, which aligns with common media frames that emphasize certain narratives about the country. This portrayal probably influences Dimas initial reaction to the news that he must go to Kyiv, as he immediately considers bribery as the norm for dealing with officials in Ukraine: ‘In der Ukraine dagegen funktioniert kein Behördengang ohne Bestechung. Aber wie ist man in Kiew korrekt korrupt? Keine Ahnung.’ (14–15) Dima’s acknowledgment of bribery as a pervasive issue in Ukrainian society adds a humorous element to the observation, reflecting a cynical perspective on the bureaucratic challenges he anticipates facing. In this phrase, the repetition of the ‘k’ sound in ‘korrekt’ and ‘korrupt’ creates a rhythmic and memorable effect, drawing attention to the contrast between the two words. The alliteration enhances the playful and satirical tone of the phrase. Moreover, Dima’s very first impressions shortly before landing rather strongly reflect well-known images of Ukraine, including corruption, post-Soviet aesthetics, orthodox churches, and environmental dangers: ‘Aus der Luft kann man die Korruption nicht erkennen. Die abgeschabten Hochhäuser, die rostigen Fabrikdächer, die leeren Swimmingpools vor den Datschas — sie könnten schließlich auch mit individuellem Mismanagement zusammenhängen. (…) Die goldenenen Kuppeln der orthodoxen Kirchen und Kloster strahlen. (…) Oh, und ein Reaktor, sicherlich nicht Tschernobyl, der trägt ja Sarkophag, trotzdem gruselig.’ (32–33)

Furthermore, Mrs Kunze’s attitude towards Ukrainian leaders should be mentioned. She commiserates with Dima about the upcoming battle with the Ukrainian red tape and then concludes: ‘Un jetzt noch der Köhmikar bei euch als Präsidend … ’ (14) For a civil servant in an official situation, calling the president of another country a comedian is disrespectful (even though Volodymyr Zelensky is an actor by trade and has appeared in numerous television comedies), as such a statement implies that the current Ukrainian president is incapable of doing his work. However, Dima does not object to this attitude. He adopts this view and refers to President Zelensky throughout the story as ‘comedian-president’. To be fair, the predecessors of Zelensky are ironically marked by Dima as ‘sweetmeats-producer’ (56) or ‘sweetmeats-president’ (62)^37^ and ‘the twice convicted one in the helicopter’ (62),^38^ respectively; that is, not very respectfully, either. The perception of Ukrainian politicians and political parties as insubstantial alliances oriented toward personal interests aligns with Germany’s historical focus on its relationship with Russia. Germany’s significant presence in Moscow, including its largest embassy, as well as its academic institutions and journalists, has influenced its perspective on Eastern European politics. This lens may lead German observers to view Ukrainian political actors through a sceptical or dismissive filter, considering them as lacking genuine political substance and primarily motivated by personal interests.^39^ It should be noted that Germany’s policies towards Moscow over the past two decades were consistent but ineffective, based on the belief that increased economic relations and dialogue, guided by the principle of avoiding provocation at all costs, would lead to success in achieving reform^40^ and Russia’s admission to the democratic European nations.^41^

Another example of Dima’s German view of Ukraine is the moment when he feels annoyed by the abundance of Ukrainian flags in Kyiv (50). In German culture, a national flag is today still associated with the parades and rituals of National Socialism, such as hoisting the flag to announce one’s support of Hitler.^42^ Therefore, national flags symbolize, from the German perspective, not patriotism but nationalism.^43^ Thus, after the processes of *Vergangeheitsbewältigung* Germans often refrain from showing their flag in private and public contexts, as it is associated with concepts such as right-wing populism and nationalism.^44^ Therefore, Dima feels annoyed at the sight of Ukrainian flags, not considering that, in the case of different nations, their flags might represent different values and that national pride does not necessarily mean (extreme) nationalism.^45^

Beside his Western-influenced German perspective on Ukraine, Dima inadvertently adopts a particular post-Soviet viewpoint. These two perspectives of his are intertwined and occasionally challenging to distinguish from one another. It must be noted that Dima’s post-Soviet perception of Ukraine seems to be primed by his parents, and can also be seen as a part of ‘implicit collective memory’. According to Erll, priming in this context means that an information/fact is seen and heard repeatedly, often without awareness, and therefore seems familiar; it might be liked, understandable, and even considered true.^46^ In the given context, priming refers to the pervasive belief that the Ukrainian state is inherently dysfunctional. As claimed by Dima’s mother Vera, the Soviet time was a glorious era when the people were better off and the Kyiv tortes tasted much better (77). At the same time, as usual in the Soviet Union, nothing functioned in Ukraine back in the Soviet time, and, accordingly, nothing functions today.^47^ Dima, influenced significantly by his parents’ recurrent assertions, also internalizes the notion that Ukraine lacks credibility as a reliable state. Therefore, when looking at Ukrainians at the Leipzig airport who show no reaction when the delay of their flight is announced, Dima states that they do what they do every day — they doubt the credibility of Ukraine, and ironically comments that growing up in the milieu of post-Soviet state scepticism, instilled in him from an early age, he should have anticipated this (8).

Hence, Dima’s perspective on Kyiv tends to emphasize negative aspects, focusing on the less appealing facets of the city, and prompting him to ponder the reasons behind his parents’ decision to leave Ukraine: ‘Sahen meine Eltern irgendwann nur noch die Roststreifen und Kotreste? Wie fühlt es sich an, ein Land innerlich so aufzugeben, dass man es verlassen will?’ (49) This view can be also exemplified by Dima’s observation of Soviet monuments that currently lie abandoned, or existing as relics of a bygone era. For example, near the Monument to the Heroes, a Soviet memorial commemorating the victory over fascism, the stone ground now exhibits rust stains and pigeon droppings. In this sombre atmosphere, Dima observes a young man who hesitates briefly before using the eternal flame meant for the fallen to light his cigarette (48–49). Dima’s critical gaze also extends to another monument, namely the Mother Homeland, (erected by the Soviets in 1981) evoking a similar sense of detachment. He raises questions regarding the symbolism of this monument, pondering whether it represents a relic of the past or contemporary issues like corruption. Moreover, he contemplates the monument’s significance in the context of the ongoing conflict in Donbas, further highlighting his reflective approach towards symbols of national identity (50).^48^ It should be noted that Dima does not explicitly harbour anti-Russian sentiments; and yet, he expresses apprehension regarding the Donbas conflict. For instance, he acknowledges his limited knowledge about the conflict but critiques the use of war exhibits as backdrops for Instagram photos featuring women in suggestive poses which implies that the reality of the war has become distant and removed from the everyday lives of people in Kyiv.^49^

To conclude, Dima’s perceptions of Ukraine before and at the outset of his journey reflect a convergence of two distinct viewpoints: the German perspective and his parents’ post-Soviet worldview. This blend leads Dima to adopt a seemingly neutral position, highlighting an anti-war or pro-peace perspective^50^ that resonates with the wish to distance him from his parents as well as with the prevailing pacifist sentiments often observed in the German mainstream media.^51^

## Change of Dima’s View

In his attempt to obtain the renewed birth certificate, Dima chooses not to be ‘correctly corrupt’. Instead, he opts to follow the ‘insane regular way first’ (42), visiting the local citizens’ registration office to acquire the necessary documentation. This choice reflects his aversion to bribery, considering it an unnatural practice he is unwilling to engage. This decision also highlights the contrast between Dima’s expectations and the realities of navigating bureaucratic processes in Ukraine.

For instance, Dima humorously expresses his apprehension towards encountering corrupt and intimidating officials in Ukrainian government offices. The description of ‘hairy, nasty fifty-year-olds in uniform’ with ‘dollar sign-shaped serpent tongues’ (44) conveys a caricatured image of corrupt bureaucrats, employing exaggerated and grotesque imagery for satirical effect. Moreover, the juxtaposition between Dima’s anticipation of encountering corrupt officials and the reality of encountering ‘three young ladies’ in office Nr 4, who steadfastly refuse any form of bribe (46), adds a layer of irony to the situation. This humorous portrayal serves to critique the stereotypes and prejudices that Dima, and potentially others, hold about East-European bureaucracy. Also, Dima’s relatives express astonishment at this kind of efficiency, highlighting their lack of accustomedness to such smooth processes in the country (46).

Initially, however, Dima’s appreciation for Ukraine is superficial, driven by the desire to obtain his papers and distance himself from his past. He still perceives the need to stay in the country (to deal with both Ukrainian and German bureaucracy) as a burden, or *golovnjak* (Russian word for a severe headache). (83)^52^ Nonetheless, the arrival of Dima’s father, Leonid, in Kyiv, his declining health, and subsequent hospitalization compel Dima to stay in Ukraine, providing care for his father while residing with family members Zoja and Andrej. This situation compels him to confront his roots and heritage more deeply, marking the beginning of a more profound exploration of his Ukrainian identity.

Unlike Dima, Leonid feels at home in the Ukrainian language and culture, and it seems that he fits very well into this environment. He suffers from memory loss, but his recollection of Soviet proverbs and quotes remains vivid (102 and 113). He finds it easy to establish contact with Zoja, Andrej, and other family members. As Dima takes on the responsibility of caring for his father, he gains a newfound closeness to him and realizes how little he knows about his parents (105). When Leonid requires hospitalization, Dima initially feels helpless. However, with the assistance of Tjotja (auntie) Jana, he is able to navigate the system. Though initially hesitant to offer a bribe for Jana’s assistance (111), he gradually comes to understand that this is more a reflection of the daily struggles within the Ukrainian healthcare system than an indication of widespread corruption (e.g. 125, 119, 129, 130 or 139). At this point, the novel vividly contrasts perceptions of Germany with those of Ukraine, presenting a paradoxical perspective. Germany is depicted as a highly organized and efficient country, yet simultaneously viewed as colder and less personal compared to Ukraine. This duality is humorously illustrated by Jana’s incredulous remark about the German health insurance system: ‘Was ist das denn für ein System, in dem ihr in Deutschland lebt? Wo man gar nichts mit persönlichen Kontakten und ein wenig Geld regeln kann? Das ist doch unmenschlich!’ (135) Conversely, Ukraine emerges as a society where personal connections and bribery are part of everyday life, but warmth and human connections hold significant value. Through such juxtaposition, the novel explores the complexities of identity, subverting simplistic East–West dichotomies. It humorously challenges readers to re-evaluate their preconceived notions about what constitutes a ‘good’ society, underscoring the importance of understanding across diverse cultural, historical, and individual contexts.

Along with changes in his attitudes, Dima’s linguistic patterns also shift. At the beginning, he speaks German (or, more exactly, Saxonian dialect) with Mrs Kunze and Russian with his parents and the people of Kyiv. Interestingly, his use of Russian language in Kyiv identifies him immediately as a foreigner, as he speaks, according to an Uber driver, ‘very nice Soviet-Russian’ (53). In this context, Soviet-Russian refers to a standardized form of the Russian language used in the former Soviet Union, which was used in educational institutions as well as in official communications, government documents, and media (TV, radio, newspapers). The Russian language spoken in modern-day Ukraine differs significantly from the Russian language spoken in Russia, as there are distinctive phonetic, grammatical, and lexical differences.^53^ Since the collapse of the Soviet Union, the Russian language has undergone changes and diverged into regional varieties, and, in Ukraine, it has also taken on new social and political connotations since 2014. Therefore, the linguistic norm of Standard Russian is nowadays less often taught to Russian speakers in Ukraine, as they mainly attend Ukrainian-language schools.^54^

Thus, Dima’s use of Soviet-Russian in Kyiv makes him feel like a foreigner, experiencing belonging and estrangement at the same time (53). For instance, he faces a language barrier while communicating with locals, as he finds it difficult to understand their jokes and cultural references; this makes him feel isolated and disconnected from the local culture (148). In addition, despite his efforts to blend in and appear like a local, Dima is often identified as a foreigner, which results in him feeling like an outsider in the country which used to be his homeland. He experiences a sense of disillusionment, as his efforts to integrate and assimilate into the local culture are viewed as inadequate or inauthentic (e.g. 137 and 148). However, as Dima begins to communicate more with his Ukrainian relatives, he becomes increasingly comfortable using the Ukrainian language. This linguistic transition not only allows him to connect with his family on a deeper level but also helps him to integrate more fully into Ukrainian culture. He gradually begins to speak like other Ukrainian men, which, to be fair, may include a significant amount of swear words (e.g. as in Andrej’s statement about former president Yanukowich’s house: ‘Die jebuchija Supervilla von unserem alten Präsidenten, nachuj.’)^55^ Swearing serves as a means for Dima to express strong emotions and establish a sense of solidarity with his male relatives around him. Concurrently, he adopts again the guise of a self-appointed mediator, humorously portraying himself as a cultural insider while simultaneously satirizing common stereotypes and norms: ‘Fluchen ist in der Ukraine eine gegenwärtige soziale Erleichterungspraxis.’ (95) Through his language use, Dima crosses a linguistic and cultural barrier, gradually assimilating local norms and values and embracing his Ukrainian identity.

Language is often utilized as a tool to encode and retrieve memories; different languages may carry distinctive cultural associations and emotional connotations that could impact how memories are stored and recollected. The manner in which an experience is described using language can significantly affect how it is remembered and processed by the brain.^56^ Language and memory are closely intertwined, and individuals often recall events or details more effectively when they are recollected in the same language they were experienced in.^57^ In Dima’s case, his re-learning of the Ukrainian language evokes memories of his childhood experiences, and his recollections of his past in Ukraine become more vivid and easily retrievable when he speaks Ukrainian (153). Thereby, Dima also starts to reconcile with his past, and, as a result, he is able to reconnect with his parents. As his mother also arrives in Kyiv and he accompanies her on errands, their conversations about the past stir up an increasing number of childhood memories. Among them, a particularly vivid recollection emerges of a day when they shared a serene and delightful experience together. They leisurely walked down Khreshchatyk, savoured ice cream, and revelled in the tunes of street musicians. In that moment, Dima envisioned his future: ‘Wenn ich groß bin, will ich Musik und überhaupt alles so genießen können wie Mama.’ (157) The contrast between those cherished memories and today’s world, where his mother’s contentment has waned, is palpable. These beautiful memories serve as a catalyst, motivating Dima to enhance his relationship with his mother. Moreover, Dima’s journey helps him overcome his ‘Kyiv complex’, which refers to his struggles in understanding his Ukrainian roots due to his unfamiliarity with life in the Soviet and post-Soviet eras. This made it difficult for him to comprehend his parents and their way of life. Now, Dima acknowledges the strength and courage his parents had to emigrate from Ukraine, which can be seen as a reflection of his own desire to move forward and search for his identity (144).

This leads to reconciliation between Dima and his parents. As his father’s health improves, they take a leisurely stroll through the city. Thereby, Dima is transported back to happier memories and experiences a desire to recapture his childhood: ‘Wie schön es jetzt wäre, kaum groß genug zu sein, um auf die Fleischauslage sehen zu können. Klein und mit unendlichem Vertrauen zu warten, bis Papa und Mama Salo gekauft haben und mit mir weitergehen. Nur für eine Minute.’ (165) This moment marks a turning point in Dima’s relationship with his parents, as he begins to come to terms with his identity: ‘So fremd wie ein Mensch hier nur sein kann, so verwurzelt, wie ein Mensch hier nur sein kann. So traurig und beseelt, wie ein Sohn mit zwei Staatsfamilienleben sein kann. Ich liebe diese verlorenste unserer Vergangenheiten, ich liebe Kiew.’ (165) It also represents coming full circle for Dima, as he finally begins to appreciate the importance of his Ukrainian roots and the significance of his parents’ sacrifices in their journey to find a better life. By embracing his past, Dima is able to move forward and start anew, forging a deeper connection with his family and his cultural heritage. Hence, *Eine Formalie in Kiew* can be regarded as a *Generationenroman*, delving into the lives and experiences of various generations within a family. While the novel does touch upon more extensive themes like immigration, belonging, and identity, these topics are predominantly examined the family’s and Dima’s intensely personal experiences rather than within a wider transnational context. Therefore, this novel can also be interpreted as a *Bildungsroman*, as it illustrates Dima’s personal growth and development throughout the narrative. In line with Erll’s concept of implicit collective memory, *Eine Formalie in Kiew* serves as a poignant exploration of intergenerational experiences within a family, encapsulating the struggles and sacrifices made by previous generations. In this way, the novel exemplifies the interplay between individual and collective memory, illustrating how personal growth and development might be intertwined with broader historical and cultural narratives.

In the initial stages of the novel, Dima’s view of Ukrainian life and society was filtered through his ‘German lens’. However, as the narrative unfolds, his perspective undergoes a transformation, leading him to critique Germany’s bureaucratic system from a ‘Ukrainian lens’. On their way back to Leipzig, Dima and his father have to wait at the passport control for four hours. In this time, Dima observes a troubling incident where an elderly Ukrainian woman, intending to visit her three children in Germany, is interrogated as if she were a criminal. She is bombarded with invasive questions about her financial status, her children’s addresses, their job positions, and even their partners (171). Moreover, the German female official calls all the children,

(…) um jedem von ihnen am Ende zu erklären, wenn sie im Vorfeld einen Extra-Antrag bei der Ausländerbehörde gestellt hätten, “bräuchte die Mama auch nicht so lange an der Kontrolle”. In meinem Kopf höre ich mich brüllen: Erstens hast DU sie gerade ewig an der Kontrolle festgehalten, nachuj, obwohl alle Papiere in Ordnung waren. Zweitens ist es nicht *die Mama*, sondern *Ihre Mutter.* Auch Nicht-Deutsche haben das verdammte Recht, gesiezt zu werden. Warum spricht sie mit ihnen, als wären sie zurückgeblieben? (171–72)

This scene starkly illustrates Dima’s transformed perspective. While he had previously voiced criticism about specific aspects of German society, such as the rise of AfD and right-wing as well as anti-Semitic sentiments in Leipzig (10, 23), his critique had been relatively polite and discreet. However, in this situation, he resorts to strong expletives akin to a genuine Ukrainian, displaying his criticism openly and without reservation. Furthermore, just as Dima receives an email notification from Mrs. Kunze confirming his approval for German citizenship, he must witness his father suffering a heart attack, with no assistance forthcoming from the Germans waiting in the queue (172–73). This incident serves as yet another illustration of Tjotja Jana’s reference to the inherent inhumanity within the German system. Thus, the Leipzig airport serves in the novel as a standardized and depersonalized ‘non-place’, evoking feelings of disorientation, disconnection, and anonymity while also providing opportunities for encounters and exchanges; it represents a space where individuals can forge new identities.^58^ It can also function as a hybrid space, showcasing the dynamic and fluid nature of contemporary environments and challenging conventional cultural dichotomies.^59^

Symbolically, as the novel draws to a close, Dima finds himself in a state of liminality, mirroring his initial predicament. A year after initiating the process of obtaining German citizenship, he remains suspended in a state of denaturalization, entangled within Ukraine’s bureaucratic complexities. Surprisingly, he seems at peace with this perpetual state of in-betweenness, residing metaphorically on a figurative ‘border’. This acceptance marks a significant transformation from his initial perspective, which intertwined German and post-Soviet viewpoints. Now, Dima navigates the world through a prism that melds both German and Ukrainian perspectives, reflecting his journey towards embracing this dual heritage.

## Conclusion

The reception of *Eine Formalie in Kiew* encompasses a spectrum of perspectives. Reviewers interpret the novel as a rich tapestry of family history spanning different epochs and societies and praise the tender portrayal of both author’s old and new homelands.^60^ Also the novel’s infusion of humour and irony, as well as innovative use of language is commended and thoroughly analysed.^61^ Amidst these diverse perspectives, the present article contextualized this novel within the framework of memory studies. By integrating the Eastern Turn and implicit collective memory approaches, it explored how Kapitelman’s narrative delves into the implicit patterns embedded within personal, familial, and collective memories. Through the lens of memory, the novel illuminates how changes in circumstance, location, and language reveal these patterns, facilitating personal evolution and growth.

Moreover, Kapitelman’s novel serves to enrich the knowledge of the German audience about Ukraine, while also deepening their understanding of Germany itself. Thereby, *Eine Formalie in Kiew* navigates between moments of poignancy and humour, using satire as a tool to interrogate societal norms and stereotypes. This approach contributes to the ‘thickening’ of memorial cultures, as different layers and scales of memory converge to enrich both German and Ukrainian perspectives, challenging ingrained beliefs and prompting readers to question societal conventions.

According to Jänchen, border crossings play a pivotal role in shaping identity in Kapitelman’s debut novel, illustrating the endeavours of intercultural characters to find their place in a narrative characterized by multiple border crossings on various levels.^62^ This theme also reverberates throughout the novel *Eine Formalie in Kiew*, in which border crossings (e.g. going to Germany at the beginning of 1990s and travelling to Kyiv years later) symbolize profound transformations. The novel charts Dima’s internal evolution and his journey towards reconciling with his past and family lineage. By the story’s conclusion, he recognizes the relative insignificance of nationalities, stating that nothing is as indifferent as nationalities (175). He embraces his Jewish-German identity, now enriched with a Ukrainian dimension.

The novel paints a multifaceted portrait of Ukraine and the divergent perspectives that exist regarding the country, illustrating how some of these divisions can be transcended through personal growth and a deeper understanding, fostering greater tolerance. As Erll highlights, this underscores the importance of recognizing the latent influence of ingrained forms of collective memory in various dimensions, such as certain thought patterns that shape our perceptions.^63^ Therefore, Kapitelman’s work serves as a timely commentary on the power of mediated opinions, emphasizing the role they play in shaping our understanding of other countries. This resonates with the author’s active engagement in social media, where he discusses political events in Germany and advocates for support to Ukraine amidst ongoing war with Russia.

## Footnotes

1 Sabine Egger, ‘Contemporary Ukrainian Writers as an “Avant-garde” of Exile in the German Literary Field? Katja Petrowskaja and Kateryna Mishchenko’, *Oxford German Studies*, 52.3 (2023), 384–98 (p. 387).

2 Egger, ‘Contemporary Ukrainian Writers as an ‘Avant-garde’ of Exile in the German Literary Field?’, p. 388.

3 Brigid Haines, ‘The Eastern Turn in Contemporary German, Swiss and Austrian Literature’, *Debatte: Journal of Contemporary Central and Eastern Europe*, 16.2 (2008), 135–49.

4 Bernhard Blumenau, ‘Breaking with Convention? *Zeitenwende* and the Traditional Pillars of German Foreign Policy’, *International Affairs*, 98.6 (2022), 1895–913.

5 Benjamin Tallis, ‘The Zeitenwende Is Here, It’s Just Unevenly Distributed’, *Internationale Politik Quarterly* (3 April 2023) <https://ip-quarterly.com/en/zeitenwende-here-its-just-unevenly-distributed> [accessed 16 October 2023]; Matthias Mader and Harald Schoen, ‘No Zeitenwende (yet): Early Assessment of German Public Opinion Toward Foreign and Defense Policy After Russia’s Invasion of Ukraine’, *Politische Vierteljahresschrift*, 64 (2023), 525–47 (pp. 542–44).

6 Egger, ‘Contemporary Ukrainian Writers as an ‘Avant-garde’ of Exile in the German Literary Field?’, p. 397.

7 Dmitrij Romashkan (Kapitelman), ‘Kapitelmans Kind. Warum mein Vater unbedingt will, dass ich wieder Jude werde  — und wie schwer es ist, diesen Wunsch tatsächlich zu erfüllen’, *taz* (7 June 2013) <https://taz.de/Judentum-und-Identitaet/!5065923/> [accessed 7 November 2022].

8 A quota refugee (‘Kontingentflüchtling’ in German) is an individual who is selected for resettlement in a foreign country based on specific quotas or numbers allocated by that country. See Jannis Panagiotidis, *Postsowjetische Migration in Deutschland. Eine Einführung* (Weinheim: Beltz Juventa, 2021), pp. 47–52.

9 See for example Dmitrij Kapitelman, ‘Werk ohne Auftrag. Wie politisch sollte Literatur sein? Ein Plädoyer für die Freiheit der Kunst’, *Jüdische Allgemeine* (8 October 2018) <https://www.juedische-allgemeine.de/kultur/werk-ohne-auftrag/> [accessed 17 October 2023].

10 ‘Ganz ohne Polemik: nach drei Monaten Krieg gegen die Ukraine, der versuchten Auslöschung eines demokratischen Landes  — weiß ich weiter nicht im Geringsten, wofür der Deutsche Bundeskanzler wirklich steht und was er will.’ (@Kapitelmanslife, 29 May 2022) [accessed 16 November 2023].

11 Annabelle Jänchen, ‘Border Crossing and Self-Placement of Characters with a Migratory Background in Contemporary German Novels’, *Journal of Frontier Studies*, 2 (2021), 85–102 (pp. 94–96).

12 Stuart Taberner, ‘Re-articulations of German-Jewish Identity in Adriana Altaras’s titos brille and Dmitrij Kapitelman’s Das Lächeln meines unsichtbaren Vaters’, *German Studies Review*, 44.2, 359–77 (p. 368).

13 Agnes C. Mueller, ‘Germans, Migration, and Holocaust Memory in Contemporary Literature’, in *The Holocaust Across Borders: Trauma, Atrocity, and Representation in Literature and Culture*, ed. by Hilene S. Flanzbaum (Lanham: Lexington Books, 2021), pp. 74–88 (p. 74).

14 Taberner, ‘Re-articulations of German-Jewish Identity’, p. 372.

15 All quotes are from Dmitrij Kapitelman, *Eine Formalie in Kiew* (Berlin: Hanser, 2021). In the following, page numbers are given in round brackets in the text.

16 Ольга Коренева Антонова and Александра Лудевиг, ‘Практика применения программы дискурсивного анализа atlas.ti: вариация немецкого этнолекта и его влияние на технику перевода мигрантской литературы’, *Речевые технологии / Speech technology*, 1 (2022), 60–82 (p. 64). Similarly, in his review, Platthaus highlights the unconventional use of the German language in Kapitelman’s novel, which is satirically accentuated, particularly through the author’s role as a bilingual speaker. See Andreas Platthaus, ‘Die Migration hört eigentlich nie auf’, *Frankfurter Allgemeine Zeitung* (03 March 2021) <https://www.faz.net/aktuell/feuilleton/buecher/rezensionen/belletristik/erzaehlung-ueber-heimkehr-nach-kiew-von-dmitrij-kapitelman-17166180.html?printPagedArticle=true#pageIndex_2> [accessed 12 March 2024].

17 Constitutional patriotism is understood as a form of political belonging centred on universalist norms but adapted for specific constitutional cultures. The idea was born in post-war West Germany and has been advanced as a way of conceptualizing political integration at the supranational level; see Jan-Werner Müller and Kim Lane Scheppele, ‘Constitutional Patriotism: An Introduction’, *International Journal of Constitutional Law*, 6.1 (2008), 67–71 (p. 68). It somewhat replaced the traditional concept of ‘patriotism’, which was regarded in post-war Germany as too close to ‘nationalism’. Today, ‘nationalism’ is in Germany still associated with protectionism, authoritarianism, and (right-wing) populism, see Marco Bitschnau and Marlene Mußotter, ‘(National) Pride and (Conceptual) Prejudice: Critical Remarks on the Distinction Between Patriotism and Nationalism’, *Journal of Political Ideologies*, (July 2022), 1–15 (pp. 2–4) <https://doi.org/10.1080/13569317.2022.2096301> [accessed 16 November 2023].

18 See Dmitrij Kapitelman, ‘Was ist Heimat? Im Camp der Bestmöglichangekommenen’, *Aus Politik und Zeitgeschichte. Zeitschrift der Bundeszentrale für politische Bildung*, 11–12 (2017), 4–8 (p. 7).

19 According to Platthaus, this perspective on language is a ‘brilliant contribution’ to the ‘so-called migrant literature’; see Platthaus, ‘Die Migration hört eigentlich nie auf’ (n.p.).

20 See for example Saša Stanišić, ‘Three Myths of Immigrant Writing: A View from Germany’, *Words Without Borders* (03 November 2008) <https://wordswithoutborders.org/read/article/2008-11/three-myths-of-immigrant-writing-a-view-from-germany/> [accessed 08 March 2024].

21 Haines, ‘The Eastern Turn in Contemporary German, Swiss and Austrian Literature’, pp. 138–39. See also Nora Isterheld, *‘In der Zugluft Europas.’ Zur deutschsprachigen Literatur russischstämmiger AutorInnen* (Bamberg: University of Bamberg Press, 2017), p. 149; Aigi Heero, ‘Zwischen Ost und West: Orte in der deutschsprachigen transkulturellen Literatur’, in *Von der nationalen zur internationalen Literatur. Transkulturelle deutschsprachige Literatur und Kultur im Zeitalter globaler Migration*, ed. by Helmut Schmitz (Amsterdam: Rodopi, 2009), pp. 205−25.

22 Michael Rothberg, ‘Multidirectional Memory in Migratory Settings: The Case of Post-Holocaust Germany’, in *Transnational Memory. Circulation, Articulation, Scales*, ed. by Chiara De Cesari and Ann Rigney (Berlin: De Gruyter, 2014), pp. 123–45 (pp. 129–30).

23 Haines, ‘The Eastern Turn in Contemporary German, Swiss and Austrian Literature’, p. 140.

24 Isterheld, *‘In der Zugluft Europas’*, pp. 182–83.

25 Astrid Erll, ‘The Hidden Power of Implicit Collective Memory’, *Memory, Mind & Media*, 1.14 (2022), 1–17 (p. 2) <https://doi.org/10.1017/mem.2022.7> [accessed 16 November 2023].

26 Erll, ‘The Hidden Power of Implicit Collective Memory’, p. 1.

27 Ibid., p. 4.

28 Ibid.

29 Dmitrij Kapitelman, ‘Wie der Streit um die Ukraine meine Familie spaltet’, *Neue Zürcher Zeitung* (10 June 2022) <https://www.nzz.ch/feuilleton/dmitrij-kapitelman-wie-der-streit-um-die-ukraine-meine-familie-spaltet-ld.1681581> [accessed 05 October 2023].

30 Andreas von Schumann and Roman Ivanov, *Ukraine Through German Eyes. Images and Perceptions of a Country in Transition* (Bonn: Deutsche Gesellschaft für Internationale Zusammenarbeit, 2018), p. 21.This research was conducted in Germany. Similar research was carried out in 2020 in Ukraine by the Ukrainian Institute. The outcome of this research by and large confirmed the results of the former. According to Husiev and Zaderei, awareness of Ukraine in Germany has increased; however, the attitude of ordinary Germans to Ukraine remains neutral or reservedly positive. However, these positive associations lack specific details and substantial knowledge. They instead convey the general feeling about the country and its people. At the same time, negative associations are related to particular phenomena and events; see Igor Husiev and Natalia Zaderei, ‘Perception of Ukraine Abroad. Germany’, *Ukrainian Institute* (2020) <https://ui.org.ua/en/sectors-en/en-projects/en-research-analytics/perception-of-ukraine-in-germany/> [accessed 17 October 2023].

31 Schumann and Ivanov, *Ukraine Through German Eyes*, p. 21–22.

32 Ibid., p. 23.

33 Ibid., p. 22. See also Stefan Meister, *Isolation and Propaganda. The Roots and Instruments of Russia’s Disinformation Campaign* (Washington, Transatlantic Academy, 2016), pp. 6–11; Yevhen Fedchenko, ‘Kremlin Propaganda: Soviet Active Measures by Other Means’, *Sõjateadlane (Estonian Journal of Military Studies)*, 2 (2016), 141–70; Susanne Spahn, ‘Russian Media in Germany. How Russian Information Warfare and Disinformation Have Affected Germany’, *ISPSW Strategy Series: Focus on Defense and International Security*, 766 (2021), 4–11.

34 Christian von Sikorski and Jörg Matthes, ‘Framing and Journalism’, *Oxford Research Encyclopedias* (28 February 2020) <https://doi.org/10.1093/acrefore/9780190228613.013.817> [accessed 18 November 2022].

35 Schumann and Ivanov, *Ukraine Through German Eyes*, pp. 30–31. Another study of the perception of the Ukrainian-EU relationship in German and Russian newspapers from 2009 to 2015 has shown that media coverage in Germany on this topic was mostly dominated by frames such as ’politics’ and ’economy’, suggesting that deepening the EU-Ukraine relationship must include huge reforms of Ukraine’s public administration and judicial sector and rather disregarded other aspects such as steps toward improving political culture. See Katharina Kleinschnitger, Michèle Knodt and Nadiya Safonova, ‘Frames and Images Facing Ukraine: Comparing Germany’s and Russia’s Media Perceptions of the EU Relations with Ukraine’, *Cognition, Communication, Discourse*, 17 (2018), 37–55 (p. 48).

36 Erll, ‘The Hidden Power of Implicit Collective Memory’, p. 7.

37 Petro Poroshenko’s company, UkrPromInvest, acquired multiple state-owned confectionery enterprises in Ukraine between 1996 and 1998. These enterprises were consolidated to form the Roshen Holding in 1996, establishing the largest confectionery manufacturing operation in Ukraine. Subsequently, Poroshenko faced allegations of corruption and nepotism. See Graham Stack, Sergei Kuznetsov and Ben Aris, ‘Poroshenko’s Empire  — the Business of Being Ukraine’s President’, *bne Intellinews* (29 August 2016) <https://www.intellinews.com/long-read-poroshenko-s-empire-the-business-of-being-ukraine-s-president-103790/> [accessed 16 November 2023].

38 Viktor Yanukovych fled in 2014 from Kyiv by his helicopter; in 2017, he was tried in absentia for high treason and abetting Russian aggression against Ukraine. In 2019 he was found guilty of high treason and was sentenced to 13 years in prison. See ‘Viktor Yanukovych’, *Britannica* <https://www.britannica.com/biography/Viktor-Yanukovych> [accessed 16 November 2023].

39 Schumann and Ivanov, *Ukraine Through German Eyes*, p. 70.

40 John Lough, *Germany’s Russia Problem. The Struggle for Balance in Europe* (Manchester: Manchester University Press, 2021), pp. 13–18.

41 It should be stressed that Germany’s relationship with Russia is complex; while many in former East Germany still see Russia as a friend due to historical ties, attitudes in West Germany are more varied. The strategy of engagement with the East, known as ‘Ostpolitik’ and championed by Willy Brandt, is credited with easing tensions during the Cold War. Chancellor Scholz, shaped by this era, emphasizes the importance of dialogue in relations with Russia. See Matthew Karnitschnig, ‘Germany’s Ukraine Policy is Incoherent for a Reason’, *Politico* (07 March 2024) <https://www.politico.eu/article/germanys-ukraine-policy-is-incoherent-for-a-reason/> [accessed 08 March 2024].

42 Karl Christian Führer, ‘“Hoist the Flag!”: Flags as a Sign of Political Consensus and Distance in the Nazi Period’, in *Private Life and Privacy in Nazi Germany*, ed. by Elizabeth Harvey, Johannes Hürter, Maiken Umbach and Andreas Wirsching (Cambridge: Cambridge University Press, 2019), pp. 156–81 (p. 163).

43 Bitschnau and Mußotter, ‘(National) Pride and (Conceptual) Prejudice’, p. 6.

44 Julia C. Becker, Anne Enders-Comberg, Ulrich Wagner, Oliver Christ and David A. Butz, ‘Beware of National Symbols. How Flags Can Threaten Intergroup Relations’, *Social Psychology* 43.1 (2012), 3–6 (p. 5).

45 Becker et al., ‘Beware of National Symbols’, p. 5–6.

46 Erll, ‘The Hidden Power of Implicit Collective Memory’, p. 2.

47 The novel utilizes a satirical metaphor involving drain covers to illustrate the post-Soviet viewpoint. Dima, shaped by childhood advice about loosely screwed drain covers (7), clings to this belief despite a lack of supporting evidence. Even upon his return to Kyiv, he continues to sidestep drain covers, humorously acknowledging the potential outdatedness of this belief (59). This metaphor symbolizes Ukraine’s facade of functionality masking pervasive corruption. Things appear to work, like the seemingly intact drain covers, but in reality, they are broken, reflecting the underlying societal corruption.

48 The monument was very controversial from the beginning; in 2015, as Ukraine outlawed Soviet symbols in the course of decommunization, the World War II monuments were not dismantled. After the beginning of the Russian invasion in February 2022, the monument has been redefined as a symbol of national resistance. See Aleksandra Klitina, ‘Motherland. Redefining — Monument Inspires Ukrainian Artists in New Exhibition’, *Kyiv Post* (25 August 2022) <https://www.kyivpost.com/ukraine-politics/motherland-redefining-monument-inspires-ukrainian-artists-in-new-exhibition.html> [accessed 28 November 2022].

49 The Russian invasion of Donbas in 2014 appears remote to Kyiv residents as well as to Dima’s friends and family members, as they seldom discuss it and demonstrate limited understanding of its complexities. See Sabine Fischer, *The Donbas Conflict. Opposing Interests and Narratives, Difficult Peace Process. SWP Research Paper 5. April 2019* (Berlin: Stiftung Wissenschaft und Politik / German Institute for International and Security Affairs, 2019), pp. 18–19; Andrew Wilson, ‘The Donbas in 2014: Explaining Civil Conflict Perhaps, but not Civil War’, *Europe-Asia Studies*, 68.4 (2016), 631–52 (pp. 639–46). Dima’s observation of people casually posing in front of war exhibits as tourist attractions underscores a detachment from the ongoing conflict.

50 In an essay, published in 2017, Kapitelman explains this stance more thoroughly. In response to his mother Vera’s statement that Crimea has always been Russian, he became agitated. His reaction, however, was not rooted in anti-Russian sentiment but in his objection to glorifying political systems, particularly totalitarian ones. Thus, his critique aligns with a democratic and humanist perspective, emphasizing his resistance to any form of political glorification, rather than being specifically anti-Russian, see Kapitelman, ‘Was ist Heimat?’, p. 6.

51 This perspective was also widespread in 2022 in the German mainstream media when the current war in Ukraine was being discussed, as Germany’s experiences during World War II have shaped people’s pacifist attitudes, emphasizing the immorality of any kind of violence, even when it is necessary to save lives or defend a country’s independence. As an example, the open letter to Chancellor Olaf Scholz can be mentioned. The letter was published on April 29, 2022, in the magazine *Emma* and was signed by 28 German intellectuals. In this letter, a warning against the World War III was issued. By the end of January 2023, 487 000 people had signed the open letter. See: ‘Der offene Brief an Kanzler Scholz’, *Emma* (29 April 2022) <https://www.emma.de/artikel/offener-brief-bundeskanzler-scholz-339463> [accessed 08 October 2023]. Still in 2024, there is a fear that supplying Ukraine with weapons like Taurus missiles could provoke Russia into retaliating against Germany. This sentiment is echoed by nearly 60 percent of Germans, reflecting a reluctance to escalate tensions with Russia. See Karnitschnig, n.p. It should be noted, however, that this kind of pacifism has also been criticized in recent times in light of Russian full scale invasion as passive and unrealistic, see Paul Dixon, ‘The Road to Hell is Paved with Good Intentions: New Cosmopolitanism and Pacifist Warriors’, *New Political Science*, 44.3 (2022), 377–95; Felix Rösch, ‘Realism, the War in the Ukraine, and the Limits of Diplomacy’, *Analyse & Kritik*, 44.2 (2022), 201–18; Andreas Umland, ‘Germany’s Russia Policy in Light of the Ukraine Conflict: Interdependence Theory and *Ostpolitik*’, *Orbis*, 66.1 (2022), 78–94. See also Anchal Vohra, ‘What Happened to German Pacifism?’, *Foreign Policy* (07 February 2023) <https://foreignpolicy.com/2023/02/07/what-happened-to-german-pacifism/> [accessed 08 March 2024].

52 It must be noted that Dima’s bilingual or trilingual proficiency often results in code-switching, a fluid transition between languages. In Kapitelman’s text, code-switching mirrors the linguistic reality, emphasizing the narrator’s polyglot environment; see Koreneva and Ludewig, ‘Практика применения программы дискурсивного анализа atlas.ti’, p. 69. See also Till Dembeck and Rolf Parr, ‘Mehrsprachige Literatur: Zur Einleitung’, in *Literatur und Mehrsprachigkeit: Ein Handbuch*, ed. by Till Dembeck and Rolf Parr. (Tübingen: Narr Francke Attempto, 2017), 9–14 (p.10).

53 Jan Patrick Zeller and Dmitri Sitchinava, ‘The Russian Language in Belarus and Ukraine’, in *The Soft Power of the Russian Language. Pluricentricity, Politics and Policies*, ed. by Arto Mustajoki, Ekaterina Protassova and Maria Yelenevskaya. (London: Routledge, 2019), 108–22 (pp. 115–19).

54 Zeller and Sitchinava, ‘The Russian Language in Belarus and Ukraine’, p. 109.

55 ‘The f***ed up super-villa of our former president, f***.’

56 Claire Kramsch, ‘Language and Culture’, *AILA Review*, 27 (2014), 30–55 (pp. 40–44).

57 Viorica Marian and Margarita Kaushanskaya, ‘Language Context Guides Memory Content’, *Psychonomic Bulletin & Review*, 14.5 (2007), 925–33 (pp. 925–26).

58 Marc Augé, *Non-Places. Introduction to an Anthology of Supermodernity*. Translated by John Howe. (London: Verso, 1995), p.103.

59 Justine Shih Pearson, *Choreographing the Airport. Field Notes from the Transit Spaces of Global Mobility* (Cham: Palgrave Pivot, 2018), pp. 45–56.

60 Sigrid Brinkmann, ‘Der Wille zum Glück, vor langer Zeit zerbröselt’, *Deutschlandfunk Kultur* (21 January 2021) <https://www.deutschlandfunkkultur.de/dmitrij-kapitelman-eine-formalie-in-kiew-der-wille-zum-100.html> [accessed 14 March 2024]; Dietmar Jacobsen, ‘Korrekt korrupt in Kiew’, *literaturkritik.de* (04 April 2021) <https://literaturkritik.de/kapitelman-eine-formalie-in-kiew,27776.html> [accessed 14 March 2024]; Tobias Becker, ‘Sogar sächseln kann er’, *Spiegel Kultur* (25 January 2021) <https://www.spiegel.de/kultur/literatur/eine-formalie-in-kiew-von-dmitrij-kapitelman-sogar-saechseln-kann-er-a-c1856d1a-75ec-424a-9d1f-583a0cf77097> [accessed 14 March 2024].

61 See Becker, ‘Sogar sächseln kann er’, (n.p.); Platthaus, ‘Die Migration hört eigentlich nie auf’ (n.p.); Koreneva and Ludewig, ‘Практика применения программы дискурсивного анализа atlas.ti’, (pp. 64–77).

62 Jänchen, ‘Border Crossing and Self-Placement of Characters with a Migratory Background in Contemporary German Novels’, p. 96.

63 Erll, ‘The Hidden Power of Implicit Collective Memory’, p. 13.

